# Effect of hemoadsorption during cardiopulmonary bypass surgery – a blinded, randomized, controlled pilot study using a novel adsorbent

**DOI:** 10.1186/s13054-016-1270-0

**Published:** 2016-04-09

**Authors:** Martin H. Bernardi, Harald Rinoesl, Klaus Dragosits, Robin Ristl, Friedrich Hoffelner, Philipp Opfermann, Christian Lamm, Falk Preißing, Dominik Wiedemann, Michael J. Hiesmayr, Andreas Spittler

**Affiliations:** Department of Cardiothoracic and Vascular Anaesthesia and Intensive Care Medicine, Medical University of Vienna, Waehringer Guertel 18-20, A-1090 Vienna, Austria; Department of Surgery, Research Laboratories, Medical University of Vienna, Lazarettgasse 14, A-1090 Vienna, Austria; Centre for Medical Statistics, Informatics and Intelligent Systems, Medical University of Vienna, Spitalgasse 23, A-1090 Vienna, Austria; Department of Cardiac Surgery, Medical University of Vienna, Waehringer Guertel 18-20, A-1090 Vienna, Austria; Core Facilities, Core Facility Flow Cytometry, Medical University of Vienna, Lazarettgasse 14, 1090 Vienna, Austria

**Keywords:** Cytokines, Cytokine storm, CytoSorb, Cardiac surgery, Cardiopulmonary bypass, Hemadsorption, Inflammation, Interleukin, High-mobility box group 1

## Abstract

**Background:**

Cardiopulmonary bypass (CPB) surgery initiates a systemic inflammatory response, which is associated with postoperative morbidity and mortality. Hemoadsorption (HA) of cytokines may suppress inflammatory responses and improve outcomes. We tested a new sorbent used for HA (CytoSorb™; CytoSorbents Europe GmbH, Berlin, Germany) installed in the CPB circuit on changes of pro- and anti-inflammatory cytokines levels, inflammation markers, and differences in patients’ perioperative course.

**Methods:**

In this first pilot trial, 37 blinded patients were undergoing elective CPB surgery at the Medical University of Vienna and were randomly assigned to HA (n = 19) or control group (n = 18). The primary outcome was differences of cytokine levels (IL-1β, IL-6, IL-18, TNF-α, and IL-10) within the first five postoperative days. We also analyzed whether we can observe any differences in *ex vivo* lipopolysaccharide (LPS)-induced TNF-α production, a reduction of high-mobility box group 1 (HMGB1), or other inflammatory markers. Additionally, measurements for fluid components, blood products, catecholamine treatment, bioelectrical impedance analysis (BIA), and 30-day mortality were analyzed.

**Results:**

We did not find differences in our primary outcome immediately following the HA treatment, although we observed differences for IL-10 24 hours after CPB (HA: median 0.3, interquartile range (IQR) 0–4.5; control: not traceable, *P* = 0.0347) and 48 hours after CPB (median 0, IQR 0–1.2 versus not traceable, *P* = 0.0185). We did not find any differences for IL-6 between both groups, and other cytokines were rarely expressed. We found differences in pretreatment levels of HMGB1 (HA: median 0, IQR 0–28.1; control: median 48.6, IQR 12.7–597.3, *P* = 0.02083) but no significant changes to post-treatment levels. No differences in inflammatory markers, fluid administration, blood substitution, catecholamines, BIA, or 30-day mortality were found.

**Conclusions:**

We did not find any reduction of the pro-inflammatory response in our patients and therefore no changes in their perioperative course. However, IL-10 showed a longer-lasting anti-inflammatory effect. The clinical impact of prolonged IL-10 needs further evaluation. We also observed strong inter-individual differences in cytokine levels; therefore, patients with an exaggerated inflammatory response to CPB need to be identified. The implementation of HA during CPB was feasible.

**Trial registration:**

ClinicalTrials.gov: NCT01879176, registration date: June 7, 2013.

## Background

Cardiopulmonary bypass (CPB) surgery initiates a systemic inflammatory response induced by extrinsic and intrinsic factors [1–3]. Monocytes and high-mobility group box 1 protein (HMGB1), a chromatin protein, encoded by the *Hmgb1* gene in humans, are important players in systemic inflammation and belong to the main producers of pro- and anti-inflammatory cytokines [4, 5]. Once activated by the extracorporeal circuit, they might lead to a dysregulation of inflammatory homeostasis and increased levels of both, pro- and anti-inflammatory plasma mediators such as tumor necrosis factor-alpha (TNF-α), interleukin-1β (IL-1β), IL-6, IL-10, and IL-18 [4, 6–9]. This strong inflammatory response induces post-surgical monocyte immunosuppression which is indicated by an impaired production of *ex vivo* lipopolysaccharide (LPS)-induced TNF-α exaggeration [10].

All of these factors may lead to a prolonged postoperative course, including a delayed weaning from mechanical ventilation, recovery of organ functions, and discharge from the intensive care unit (ICU). Thus, measures to decrease the inflammatory process have the potential to improve the perioperative course [11]. Hemoadsorption (HA) using the CytoSorb™ adsorber (CytoSorbents Europe GmbH, Berlin, Germany) is a recent technology that has shown rapid elimination of many key cytokines that cannot be filtered by using current blood purification techniques [12].

The primary aim of this first single-center, blinded, randomized, and controlled pilot study was to investigate differences of pro- and anti-inflammatory cytokines in patients undergoing cardiac surgery with CPB using the CytoSorb™ adsorber compared with a control group within the first 5 postoperative days (POD). Furthermore, we investigated whether we can observe any differences in *ex vivo* LPS-induced TNF-α production, a reduction of HMGB1, or other inflammatory markers. Also, we investigated differences in fluid management or the use of catecholamines and differences in edema formation as determined by analysis of body composition by bioelectrical impedance analysis (BIA). Additionally, we compared length of ICU stay, respirator therapy, and 30-day mortality.

## Methods

### Ethics approval

This study was approved by the ethics committee of the Medical University of Vienna with reference number EK Nr: 1095/2013. Furthermore, we reported the study to the Austrian Federal Office for Safety in Health Care (INS-621000-0505) and registered it at ClinicalTrials.gov (NCT01879176) before recruitment started. Written informed consent to participate and consent to publish were obtained from each patient.

### Study design and patients

This study was a randomized, blinded (in patients), controlled, single-center trial in 46 adult patients undergoing elective open heart surgery (coronary artery bypass graft [CABG], valve surgery, combined procedure) with an expected CPB duration of more than 120 minutes at the Department of Cardiac Surgery, Medical University of Vienna, Vienna, Austria. The study was conducted between Sept. 10, 2013, and May 6, 2015, at our department.

**Fig. 1:**
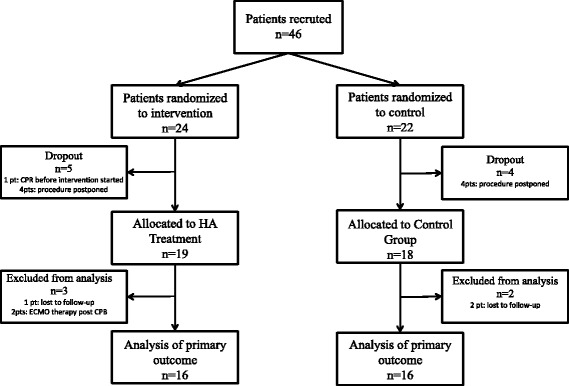
The selection process for patients included in the study

We excluded the following interventions or conditions: declined informed consent, transplant surgery, scheduled insertion of a cardiac assist device, thrombendarterectomy of the pulmonary arteries, emergency and urgent procedures, serum creatinine of more than 2 mg/dl, C-reactive protein (CRP) of more than 2 mg/dl, bilirubin of more than 2 mg/dl, body mass index (BMI) of less than 18 kg/m^2^, pregnancy, history of stroke, and patients receiving chemotherapy, anti-leukocyte drugs, TNF-α blockers, immunosuppressive drugs (e.g., tocilizumab) or with any diagnosed disease state that has produced leukopenia (e.g., acquired immune deficiency syndrome). Patient selection is shown in Fig. 1.

### Randomization

Eligible patients were enrolled the day before surgery by one of the physicians involved in the study and randomly assigned into one of two groups (HA or control). Randomization was performed as block randomization by the online Randomizer for Clinical Trials 1.7.0 (https://www.meduniwien.ac.at/randomizer). To create homogenous and comparable groups, the randomization was stratified by sex and procedures.

### Outcomes

#### Primary outcome

Primary outcomes were differences in the evolution of cytokines using the CytoSorb™ adsorber for HA during cardiopulmonary bypass.

#### Secondary outcomes

Secondary outcomes were differences in LPS-induced release of TNF-α; differences in the expression of HMGB1; changes in serum CRP or procalcitonin (PCT) concentrations; differences in the need of fluid components (crystalloid and colloid solutions), blood products (erythrocytes, fresh frozen plasma, and platelets), or catecholamine treatment; and changes in BIA, length of ICU stay, and 30-day mortality.

### Number of patients

The fact that this is the first randomized controlled study and no prior data to cytokine level alterations in cardiac surgery patients using this HA device were available made us consider a mean difference of one standard deviation between groups as a clinically relevant effect. Under this assumption, we calculated by using a *t* test a total of 16 individuals per group that are required to achieve 80 % power with a significance level of 5 %. Therefore, we planned a total of 40 patients to allow an adequately powered analysis with 20 % dropouts for complicated intraoperative course. To avoid the risk of low power, we increased the number of patients to 46 after the completion of the 36^th^ patient because a total of 7 patients dropped out at this time.

### Data collection

Preoperative patient data (age, weight, height, sex, BIA, European system for cardiac operative risk evaluation [EuroSCORE], diagnosis, preoperative myocardial infarction within 24 hours [MCI], history of asthma bronchiale, chronic obstructive pulmonary disease [COPD], insulin or non-insulin-dependent diabetes mellitus [IDDM, NIDDM], history of chronic kidney disease [CKD], dialysis, left ventricular ejection fraction [LVEF], stable and unstable angina pectoris, cardial decompensation, peripheral arterial obstructive disease [PAOD], and arterial hypertension), surgery-related factors (kind of operation, duration of anaesthesia and surgery duration of CPB and aortic cross-clamp [AoCC], unplanned insertion of assist devices, amount of fluids [cristalloids or colloids], need of catecholamines [noradrenalin, dobutamin, levosimendan, vasopressin, or milrinon], and need of blood products [erythrocytes, fresh frozen plasma, or thrombocytes] or coagulation factors [fibrinogen, prothrombin complex concentrate, desmopressin, or recombinant factor VIIa]), intraoperative diuresis and postoperative data (use of catecholamines, BIA, length of stay on intensive care unit [LOS-ICU], length of mechanical ventilation, or need of extracorporeal membrane oxygenation [ECMO]) were collected by a case report form.

### Procedure

Anaesthesia was induced and CPB circuit was primed (1000 ml crystalloid and 500 ml colloid solution together with 5000 IE heparin, and 100 ml mannitol 20 %) in accordance with institutional standards. CPB was performed by using non-pulsatile flow at 2.5 l · min^−1^ · m^−2^, a non-heparin-coated circuit, and a membrane oxygenator (Quadrox™, Maquet, Hirrlingen, Germany, or Capiox, Terumo, Eschborn, Germany). All study cases were performed by experienced cardiac anaesthesia fellows supervised by senior cardiac anaesthesiologists, both trained in transesophageal echocardiography (TOE), which was used to monitor myocardial performance and the impact of fluid loading and inotropic support on left and right ventricular function. Blood transfusion was performed in accordance with Society of Thoracic Surgeons/Society of Cardiovascular Anesthesiologists (STS-SCA) transfusion guidelines [13, 14], and administration of coagulation factors was based predominantly on rotational thromboelastometry (ROTEM) variables and the coagulation profile of each patient.

In the intervention group, we installed the 300 ml CytoSorb™ adsorber on the CPB machine. The active component of the CytoSorb™ device consists of adsorbent polymer beads composed of porous polymerized divinylbenzene. These beads have pores that can adsorb hydrophobic molecules in a size range of approximately 10 to 55 kD, which is sufficient to remove almost all known cytokines. The polymer beads are encased in a polycarbonate canister commonly used in commercially available dialyzers. Blood was pumped actively through the CytoSorb™ cartridge by using a side arm coming from the venous outflow tube and given back to the venous reservoir prior to the oxygenator. The flow through the cartridge was controlled by a roller pump with 200 ml/min to standardize flow conditions in all treated patients. The addition of 20 ml crystalloid solution was necessary to fill the additional line in the treatment group. The control group was treated similarly, but no adsorber was installed.

### Blood sampling

Blood samples were drawn in pyrogen-free vials, and plasma was separated by centrifugation and frozen (−80 °C). Blood samples for cytokines (IL-1β, IL-6, IL-18, TNF-α, and IL-10) were determined at the following time points: A, before induction of anesthesia; B, before CPB; C, at the end of CPB; D, 2 hours after CPB; E, 24 hours after CPB; F, 48 hours after CPB; and G, 120 hours after CPB. The *ex vivo* LPS-induced TNF-α production was measured at the time points A–C, F, and G; and HMGB1 at time points B, D, and E. For the quantification of IL-1β, IL-6, TNF-α, and IL-10, we used the BD™ Cytometric Bead Array (CBA) Human Inflammatory Cytokines (BD Biosciences Europe, Erembodegem, Belgium) Kit; for quantification of IL-18, Human IL-18 Instant, ELISA (eBioscience, Inc., San Diego, CA, USA), and for quantification of HMGB1 the high-mobility group box 1 (HMGB1), ELISA Kit (MyBioSource, Inc., San Diego, CA, USA). For the measurement of *ex vivo* LPS-induced TNF-α, lipopolysaccharide from *Escherichia coli* was purchased (Sigma-Aldrich GmbH, Vienna, Austria) and prepared. For the analysis of LPS-induced TNF-α release, Human TNF-α Instant, ELISA (eBioscience, Inc.) was performed on each sample. All analysis were conducted in accordance with the protocol of the manufacturer.

Blood samples for CRP, procalcitonin, albumine, fibrinogen, hemoglobin, thrombocytes, and leukocytes were determined at the following time points: a baseline value within 24 hours preoperatively (BL), 1st postoperative morning (1.POD), 2nd postoperative morning (2.POD), and 5th postoperative morning (5.POD).

### Bioelectrical impedance analysis

We performed BIA by using 800 μA at 50 kHz with a single-frequency bioimpedance analyzer (Model BIA 101; Akern-RJL, Pontassieve, Italy). The skin was cleaned, and adhesive pregelled electrodes (Bianostic AT; Data-Input GmbH, Wedemark, Germany) were placed on the hand (source on the third metacarpophalangeal joint and the detector on wrist, between the distal prominences of the radius and ulna) and the foot (source on the third metatarsophalangeal joint and the detector on the ankle, between the medial and lateral malleoli) of the right side while patients where in a recumbent position with the limbs abducted from the body. Measurements were performed within 24 hours preoperatively, 1.POD, 2.POD, and 5.POD. The measured BIA variables were resistance (R), reactance (Xc), the phase angle (arctanXc/R), and total body water (TBW). TBW was calculated by the following formulas according to the BIA analyzer we used [15, 16]:

$$ \begin{array}{l}\mathrm{Female}:\ \mathrm{T}\mathrm{B}\mathrm{W} = 0.382*\left(\mathrm{H}{\mathrm{t}}^2/\mathrm{R}\right) + 0.105*\mathrm{weight} + 8.315\hfill \\ {}\mathrm{Male}:\ \mathrm{T}\mathrm{B}\mathrm{W} = 0.396*\left(\mathrm{H}{\mathrm{t}}^2/\mathrm{R}\right) + 0.143*\mathrm{weight} + 8.399.\hfill \end{array} $$

### Statistical analysis

Demographic and clinical baseline data were summarized by mean and standard deviation or mean and range, expressed through minimum and maximum, for metric variables or absolute frequencies for categorical variables. Differences between groups were analyzed by using the Student’s *t* test for continuous variables and Fisher’s exact test for categorical variables.

The distributions of the cytokine levels were highly skewed; therefore, between-group differences of these variables were assessed by using the non-parametric Wilcoxon rank-sum test. The distributions were described by median and interquartile range (IQR) expressed through the first quartile and the third quartile.

The distributions of laboratory values were largely symmetric without severe outliers. These variables were described by mean and standard deviation, and the effect of HA was assessed by using analysis of covariance (ANCOVA) models. In these models, the outcome is explained by the treatment group (HA versus control), the stratification variables (procedure and sex), and the observed preoperative baseline value (except when analyzing the baseline differences). To describe the correlation between cytokine levels and duration of procedure, we calculated Spearman’s rank correlation coefficients at the end of treatment time (time point C).

The analysis of IL-10 suggests that the decrease after HA may follow an exponential function. To investigate this, we fitted the following model for time-dependent decay of IL-10 for each group by using the non-linear least squares method: mean IL-10 = A*exp(λ*time). Also, to obtain a robust global test, we used a re-randomization test with 10,000 repeats. The patients were repeatedly randomly assigned anew with the same block randomization procedure as originally applied. In each repeat, a chi-squared-type statistic and a *P* value were calculated as the proportion of resampled statistics being equal to or larger than the observed statistic.

## Results

In total, 46 patients were included in the study and randomly assigned into one of two groups (HA or control). Nine patients (5 HA and 4 control) dropped out of the study after randomization: In six patients, the procedure was scheduled to another day or later in the day, when the study team was no longer available. One patient (HA) evolved a hemodynamical instability after skin incision leading to cardiopulmonary resuscitation and an acute onset of the CPB, so no adsorber could be installed, and in two patients (1 HA and 1 control) we lost the follow-up of our main outcome measurements, so we excluded them too. Finally, we analyzed 37 patients; 19 patients were randomly assigned to the HA group and 18 patients to the control group. For the analysis of our primary outcome, we excluded the patients with unexpected post-treatment ECMO therapy (n = 2) because of differences in cytokine exaggeration (Fig. 1). Our patients had a mean age of 66 ± 12 years, the mean EuroSCORE was 5.4, 30 % of them were female, and the mean temperature during CPB was 33 ± 2 °C. All patients survived the 30-day period, except one patient (HA) who died on the 22^nd^ postoperative day because of multiple surgical complications. All other pre-, intra-, and post-operative patient characteristics showed no difference between both groups. Detailed results are shown in Table 1.

**Table 1:** Patient and surgical characteristics

	HA (n = 19)	Control (n = 18)	OR (95 % CI)	*P* value
Preoperative characteristics				
Age, years	64 (30–81)	69 (51–81)		0.1737
Male	12	14	0.50 (0.09, 2.56)	0.4756
Female	7	4		
BMI, kg/m^2^	27 (18–35)	27 (20–39)		0.6593
EuroSCORE	4.0 ± 3.6	6.0 ± 4.6		0.154
Resistance	411.7 ± 210.2	442.7 ± 101.6		0.6948
Phase angle	5.1 ± 1.5	4.9 ± 0.7		0.7078
TBW	56.3 ± 14.1	46.6 ± 11.3		0.1205
MCI	0	0		1
Asthma	0	0		1
COPD	3	3	0.94 (0.11–8.15)	1
NIDDM	6	4	1.60 (0.30–9.56)	0.714
IDDM	0	2	0 (0, 4.99)	0.2297
CKD	0	0		1
Cardiac decompensation	2	1	1.96 (0.09–124.72)	1
PAOD	3	1	3.10 (0.22–176.81)	0.6039
Art. hypertension	10	10	0.89 (0.20–3.90)	1
Dialysis	0	0		1
Angina pectoris (absence of)	16	13		0.5392
Angina pectoris (stable)	3	4		
Angina pectoris (instable)	0	1		
LVEF >50 %	12	13		0.4756
LVEF 30–50 %	7	4		
LVEF <30 %	0	1		
Intraoperative characteristics				
Valve procedure, M/F	7/4	7/4		0.4819
CABG, M/F	2/1	5/0		
Combined procedure, M/F	3/2	2/0		
Anesthesia time, min	474 (290–673)	427 (277–570)		0.1683
Surgery time, min	369 (219–630)	327 (225–493)		0.2152
CPB time, min	191 (112–288)	170 (83–274)		0.2064
AoCC time, min	138 (57–242)	117 (36–179)		0.1423
Fibrinogen, g	1.7 (0–5)	0.8 (0–4)		0.077
Thrombocytes, units	0.15 (0–1)	0.2 (0–1)		0.6304
PCC, IE	605 (0–2000)	389 (0–3000)		0.415
FFP, units	0.5 (0–9)	0		0.3306
Erythrocytes, units	1.4 (0–7)	0.7 (0–3)		0.2021
Cristalloids, ml	5239 ± 1569	4311 ± 1230		0.0526
Colloids, ml	571 ± 327	514 ± 130		0.4873
Diuresis	826 ± 352	978 ± 589		0.3516
Postoperative characteristics				
LOS-ICU, days	2.3 ± 2.0	2.4 ± 1.9		0.8721
Mechanical ventilation, days	0.7 ± 1.6	0.2 ± 0.4		0.19
Balance 1POD	9374 ± 4785	7303 ± 2664		0.1124
vaECMO, n	2	0		0.4865
30-day mortality	1	0		1

Values are presented as number (n), percentage (%), mean (range), mean ± standard deviation (SD), or odds ratio (95 % confidence interval). The listed *P* values of statistical tests were calculated by using *t* test for continuous and the Fisher’s exact test for categorical variables. Abbreviations: AP, angina pectoris; aHTN, arterial hypertension; AoCC, aortic cross-clamp; BMI, body mass index; CABG, coronary artery bypass grafting; CKD, chronic kidney disease; COPD, chronic obstructive pulmonary disease; CPB, cardiopulmonary bypass; HA, hemoadsorption; IDDM, insulin-dependent diabetes mellitus; LOS-ICU, length of stay in intensive care unit; LVEF, left ventricular ejection fraction; MCI, myocardial infarction; NIDDM, non-insulin-dependent diabetes mellitus; OR, odds ratio; PAOD, peripheral artery occlusive disease; PCC, prothrombin complex concentrate; TBW, total body water; vaECMO, veno-arterial extra corporal membrane oxygenation

### Primary outcome

We measured high amounts of IL-6 in both groups increasing after CPB and with a peak value 2 hours after CPB (HA: median 120.8, IQR 49.0–160.8 versus control: median 118.7, IQR 68.4–255.9, pg/ml, *P* = 0.6781). One patient (control) showed an increase of IL-6 after skin incision (2.1 pg/ml); in all other patients, the activation started during CPB. No significant difference was found between the treatment and control group for all time points. The correlations for IL-6 and the end of treatment duration were 0.34 for the HA group and 0.46 for the control group.

For IL-10, we observed an increase in one patient (HA) after inducing anesthesia (11.3 pg/ml). In four patients (2 HA and 2 control), the activation of IL-10 started before CPB; one of those also had an increase of IL-6. We did not find any similarities in those patients with pre-CPB increased levels of cytokines. IL-10 reached a peak value at the end of CPB (HA: median 13.1, IQR 3.3–18.7 versus control: median 18.5, IQR 5.7–68.0 pg/ml, *P* = 0.1562). The decrease of IL-10 seems to be earlier in the control group showing significant differences 24 hours after CPB (HA: median 0.3, IQR 0–4.5 pg/ml versus control: median 0, *P* = 0.0347) and 48 hours after CPB (HA: median 0, IQR 0–1.2 pg/ml versus control: not traceable, *P* = 0.0185). The correlations for IL-10 and the end of treatment time were 0.02 for the HA group and 0.32 for the control group.

**Fig. 2:**
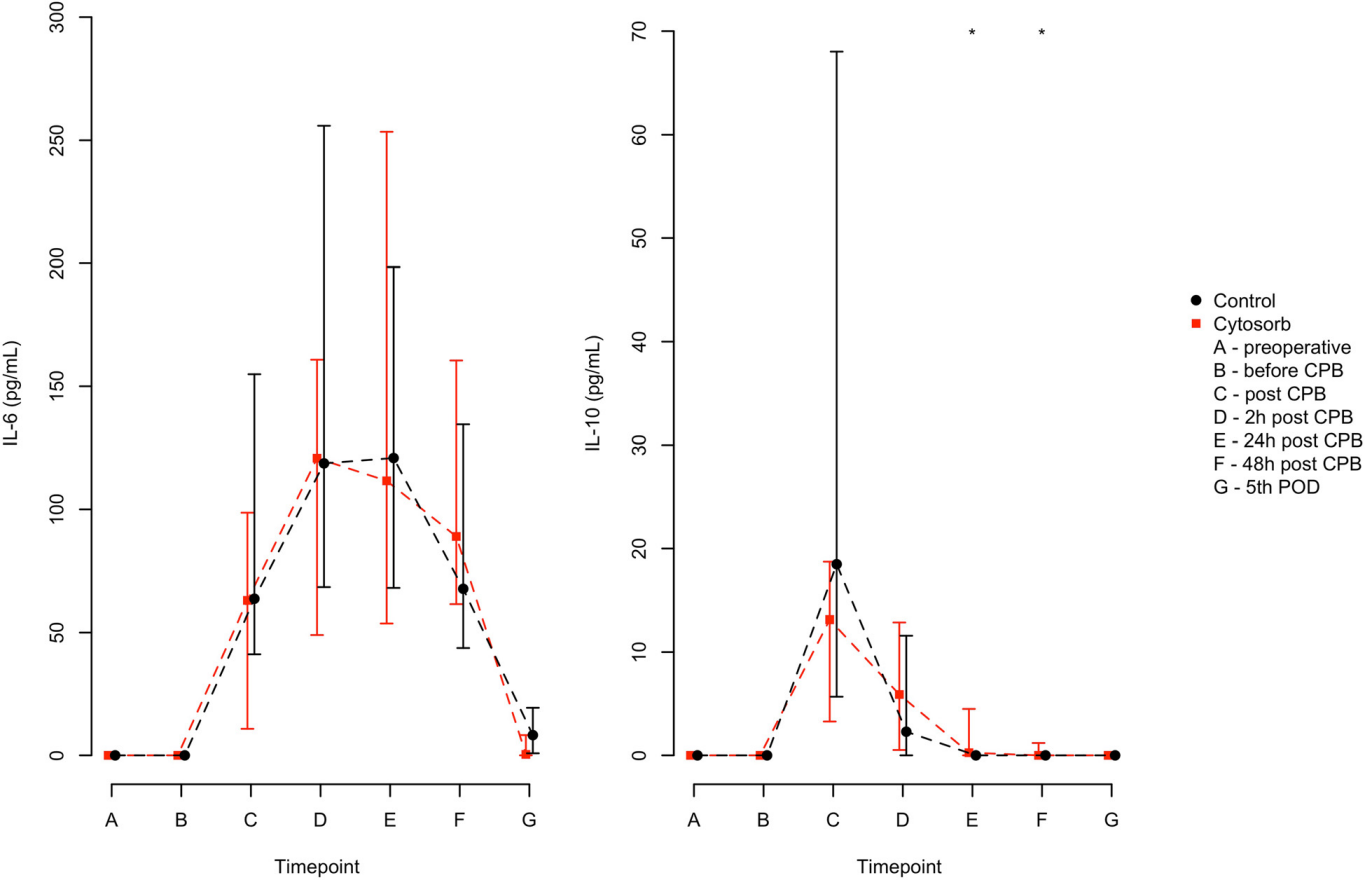
Comparison of median cytokine levels in picograms per milliliter. Red lines indicate the patients in the CytoSorb™ treatment group. Black lines indicate the patients in the control group. Error bars correspond to interquartile ranges (first quartile, third quartile). Asterisks mark differences between both groups at a significance *P* < 0.05

**Fig. 3:**
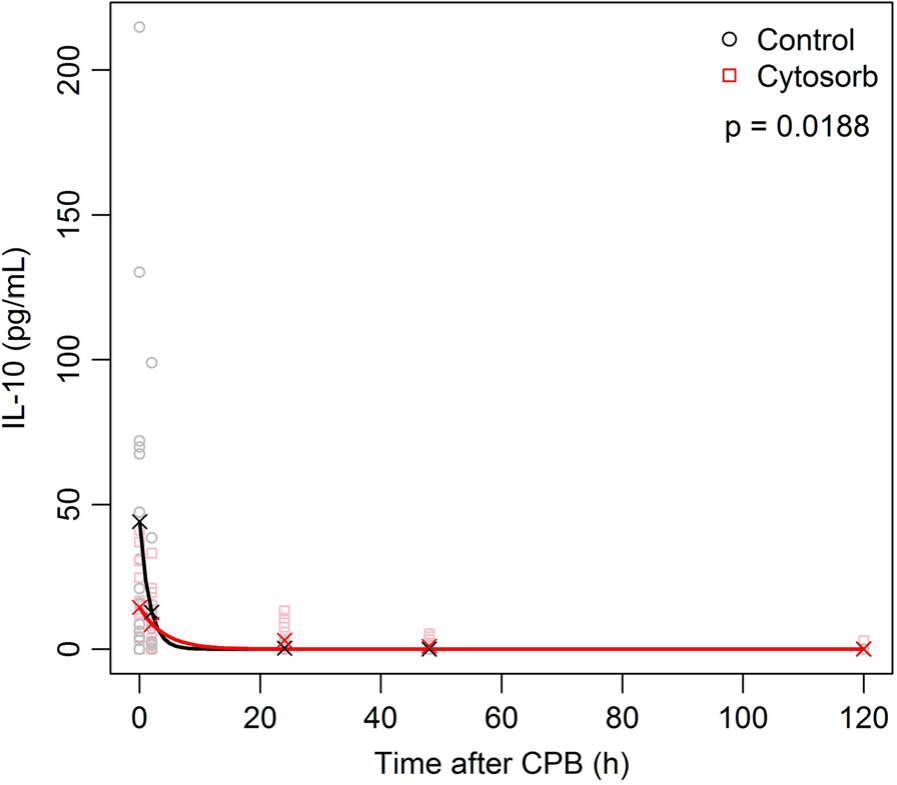
Exponential decay model for mean interleukin-10 (IL-10). Black circles indicate IL-10 values for the control group, and red squares indicate IL-10 values for the CytoSorb™ group

The exponential decay model matched rather well the observed mean values and showed different characteristics for the two groups (*P* = 0.0188). For detailed results of the analysis of the cytokine evolution, see Table 2 and Figs. 2 and 3.

**Table 2:** Comparison of cytokine levels

	Treatment	Preoperative	Before CPB	After CPB	2 hours after CPB	24 hours after CPB	48 hours after CPB	5.POD
IL-6, pg/ml	HA	Not traceable	Not traceable	62.9 (10.8, 98.7)	120.8 (49.0, 160.8)	111.6 (53.7, 253.5)	89.0 (61.4, 160.5)	0.4 (0, 8.3)
	Control	Not traceable	0	63.6 (41.2, 154.9)	118.7 (68.4, 255.9)	120.9 (68.0, 198.5)	67.7 (43.7, 134.5)	8.2 (0.8, 19.4)
	*P* value		0.3485	0.3267	0.6781	0.9837	0.3809	0.0999
IL-10, pg/ml	HA	0	0	13.1 (3.3, 18.7)	5.9 (0.5, 12.9)	0.3 (0, 4.5)	0 (0, 1.2)	0
	Control	Not traceable	0	18.5 (5.7, 68.0)	2.3 (0, 11.6)	0	Not traceable	Not traceable
	*P* value	0.3485	1.0	0.1562	0.6743	0.0347	0.0185	0.3485
TNFα-LPS, pg/ml	HA	2216 (1742, 2659)	2534 (914, 3023)	40 (1, 82)	Non fec.	Non fec.	788 (679, 1272)	1737 (843, 2535)
	Control	3364 (2579, 4893)	3403 (1679, 4394)	77 (34, 316)	Non fec.	Non fec.	3959 (2088, 4777)	3358 (3017, 3672)
	*P* value	0.004	0.1378	0.2049			0.0115	0.0205
HMGB1, pg/ml	HA	Non fec.	0 (0, 28.1)	Non fec.	5.7 (0, 34.2)	0 (0, 72.5)	Non fec.	Non fec.
	Control	Non fec.	48.6 (12.7, 597.3)	Non fec.	41.5 (4.5, 266.9)	26.2 (0, 281,2)	Non fec.	Non fec.
	*P* value		0.0208		0.1327	0.3724		

Values are presented as median (first quartile, third quartile). The listed *P* values were calculated by using Wilcoxon rank-sum tests. Abbreviations: CPB, cardiopulmonary bypass; HA, hemoadsorption; HMBG1, high-mobility group box 1; IL, interleukin; fec., fecit; POD, postoperative day; TNF-α, tumor necrosis factor-alpha; TNFα-LPS, lipopolysaccharide-induced TNF-α

We detected traceable values in only two patients for TNF-α and IL-18 and in one patient for IL-1β over the perioperative period. Therefore, we did not perform any statistical analysis on TNF-α, IL-18, and IL-1β.

Owing to technical problems with thawing of our frozen blood samples, we additionally lost one patient in the HA group and two patients in the control group. So we were able to analyze our primary outcome in only 16 patients in the HA group and 16 patients in the control group (Fig. 1).

### Secondary outcome parameters

*Ex vivo* LPS-induced TNF-α exaggeration could be stimulated at all determined time points. We observed a significant difference between both groups preoperatively (HA: median 2216, IQR 1742–2659 versus control: median 3364, IQR 2579–4893 pg/ml, *P* = 0.004) and a reduction of LPS-induced TNF-α after CPB but no significant difference between both groups. On the 2.POD, LPS-induced TNF-α reached again preoperative values but showed a significant lower amount in the HA group (HA: median 788, IQR 679–1272 versus control: median 3959, IQR 2088–4777 pg/ml, *P* = 0.0115) as well as on 5.POD (HA: median 1737, IQR 843–2535 versus control: median 3358, IQR 3017–3672 pg/ml, *P* = 0.0205). Unfortunately, we were able to analyze only 18 (9 HA and 9 control) patients on 2.POD and 17 (9 HA and 8 control) patients on 5.POD (Table 2).

HMGB1 showed a significantly different expression at baseline before treatment (HA: median 0, IQR 0–28.1 versus control: median 48.6, IQR 12.7–597.3 pg/ml, *P* = 0.0208) but no differences in the period after CPB, although post-treatment maximum levels in the control group were nearly double that of the HA group (HA: 705 versus control: 1594 pg/ml). The *post hoc* analysis was possible in 29 patients (15 HA and 14 control). We did not observe any differences in other inflammation markers like CRP or PCT or differences in leukocytes, thrombocytes, hemoglobin, albumin, or fibrinogen levels. The analysis of differences before and after intervention within the groups resulted in significant decreases in hemoglobin, albumin, and thrombocytes, according to hemodilution, as well as significant increases in CRP and leukocytes, according to usual postoperative inflammation, within both groups. We did not find differences in the change of PCT, HMGB1, or fibrinogen levels within both groups. Detailed information is shown in Tables 3 and 4.

**Table 3:** Comparison of laboratory values

	Treatment	Time point							
		preoperative	*P* value	1.POD	*P* value	2.POD	*P* value	5.POD	*P* value
CRP, mg/dl	HA	0.28 ± 0.2		6.29 ± 2.9		16.94 ± 5.3		10.09 ± 6.4	
	Control	0.19 ± 0.2	0.0966	7.01 ± 3.6	0.8779	15.38 ± 5.9	0.7084	6.36 ± 2.9	0.1438
Leukocytes, G/l	HA	7.4 ± 2.5		11.7 ± 3.7		11.8 ± 4.2		10.1 ± 3.3	
	Control	6.7 ± 1.9	0.2905	12.6 ± 2.9	0.2621	12.7 ± 2.6	0.0935	8.9 ± 2.5	0.8714
PCT, ng/ml	HA	0.12 ± 0.34		1.21 ± 2.53		1.69 ± 5.18		0.54 ± 1.10	
	Control	0.05 ± 0.03	0.5120	4.91 ± 16.20	0.3120	4.46 ± 15.66	0.4018	1.21 ± 3.57	0.3486
Hemoglobin, g/dl	HA	13.6 ± 1.5		10.9 ± 1.2		10.1 ± 1.2		9.9 ± 1.4	
	Control	13.5 ± 1.4	0.3473	10.9 ± 1.1	0.8562	10.0 ± 0.8	0.9085	9.5 ± 1.1	0.4339
Albumin, g/l	HA	41.9 ± 9.7		31.3 ± 15.6		29.0 ± 3.1		30.7 ± 3.5	
	Control	41.1 ± 3.3	0.8469	28.5 ± 3.2	0.5794	28.5 ± 4.7	0.7626	28.8 ± 3.6	0.5618
Fibrinogen, mg/dl	HA	354.5 ± 51.3		319.7 ± 72.5		500.5 ± 125.8		605.7 ± 170.4	
	Control	332.2 ± 72.2	0.2491	325.4 ± 61.0	0.8966	496.8 ± 84.2	0.7786	553.6 ± 105.0	0.1542
Thrombocytes, G/l	HA	229.4 ± 50.9		110.5 ± 45.7		101.2 ± 47.6		184.1 ± 102.5	
	Control	206.8 ± 97.4	0.2275	116.9 ± 46.9	0.3343	102.0 ± 46.6	0.9807	178.8 ± 99.5	0.9652

Values are presented as mean ± standard deviation. The listed *P* values of statistical tests were calculated by using analysis of covariance. Abbreviations: CRP, C-reactive protein; HA, hemoadsorption; PCT, procalcitonin; POD, postoperative day

**Table 4:** Differences between preoperative and postoperative values

	Treatment	Mean difference	95 % CI	*P* value
CRP, mg/dl	HA	6.0	−7.4, −4.6	<0.0001
	Control	6.8	−8.6, 5.0	0.0002
Leukocytes, G/l	HA	4.24	−5.82, −2.66	0.0115
	Control	5.86	−7.47, −4.25	<0.0001
PCT, ng/ml	HA	1.08	−2.24, 0.07	0.0649
	Control	5.07	−13.64, 3.49	0.2271
Hemoglobin, g/dl	HA	−2.7	2.0, 3.3	<0.0001
	Control	−2.7	2.0, 3.4	<0.0001
Albumin, g/l	HA	−10.5	2.1, 19.0	0.0175
	Control	−12.6	11.2, 14.0	<0.0001
Fibrinogen, mg/dl	HA	−34.8	−1.7, 71.3	0.0607
	Control	−6.8	−22.6, 36.2	0.6332
Thrombocytes, G/l	HA	−119.0	99.2, 138.7	0.0006
	Control	−89.8	60.0, 119.7	<0.0001
HMGB 1, pg/ml	HA	9.5	−38.3, 57.3	0.6764
	Control	64.2	−70.9, 199.2	0.3235

Values are presented as mean difference and 95 % confidence interval (CI). The listed *P* values of statistical tests were calculated by using paired *t* tests. Abbreviations: CRP, C-reactive protein; HA, hemoadsorption; HMGB1, high-mobility box group; PCT, procalcitonin

We performed a BIA in 19 patients (9 HA and 10 control). However no differences in baseline values in resistance, phase angle, or TBW were observed between both groups (Table 5).

**Table 5:** Comparison of bioelectrical impedance analysis

	Treatment	Time point							
		preoperative	*P* value	1.POD	*P* value	2.POD	*P* value	5.POD	*P* value
Resistance, Ω	HA	411.7 ± 210.2		265.2 ± 38.8		250.9 ± 44.0		279.6 ± 55.0	
	Control	442.7 ± 101.5	0.6090	276.9 ± 88.3	0.9240	279.0 ± 86.7	0.5111	362.0 ± 232.8	0.3355
Phase angle, °	HA	5.1 ± 1.5		3.3 ± 1.3		2.2 ± 0.9		2.9 ± 1.1	
	Control	4.9 ± 0.7	0.4731	3.5 ± 1.3	0.6025	2.8 ± 1.1	0.3138	2.7 ± 1.2	0.8895
TBW, %	HA	56.3 ± 14.1		69.3 ± 12.3		72.2 ± 12.5		66.9 ± 10.7	
	Control	46.6 ± 11.3	0.0966	65.0 ± 18.6	0.7449	63.9 ± 17.0	0.5622	56.8 ± 16.7	0.6776

Values are presented as mean ± standard deviation. The listed *P* values of statistical tests were calculated by using analysis of covariance. Abbreviations: HA, hemoadsorption; POD, postoperative day; TBW, total body water

We also analyzed the need of catecholamines in both groups within the first 24 hours, but we did not analyze differences on day 2 or 5, because only 20 patients (9 HA and 11 control) remained in the ICU after 24 hours. We did not find any differences in the need of noradrenalin, dobutamin, or levosimendan, although it is worth mentioning that only a total of eight patients received dobutamin in the control group and 11 patients in the HA group, as well as only eight (5 HA and 3 control) patients were treated with levosimendan (Table 6).

**Table 6:** Catecholamine treatment

	Treatment		Time point							
			before CPB	*P* value	After CPB	*P* value	2 hours after CPB	*P* value	1.POD	*P* value
Noradrenalin, μg kg^-1^ min^-1^	Control	Mean ± SD	0.03 ± 0.04		0.13 ± 0.11		0.08 ± 0.08		0.06 ± 0.10	
		Max	0.12		0.36		0.30		0.36	
		N	13		18		16		8	
	HA	Mean ± SD	0.04 ± 0.05		0.13 ± 0.15		0.14 ± 0.20		0.07 ± 0.14	
		Max	0.18		0.62		0.74		0.6	
		N	14	0.3138	18	0.8503	14	0.3075	7	0.8771
Dobutamin, μg kg^-1^ min^-1^	Control	Mean ± SD	0.15 ± 0.50		1.78 ± 2.38		1.50 ± 2.05		1.18 ± 1.64	
		Max	1.72		6.94		5.55		5.00	
		N	3		8		8		8	
	HA	Mean ± SD	0.05 ± 0.23		2.30 ± 2.42		1.28 ± 1.51		1.32 ± 1.58	
		Max	1.00		8.00		4.1		5.34	
		N	1	0.4163	11	0.5136	10	0.7134	10	0.7871
Levosimendan, μg kg^-1^ min^-1^	Control	Mean ± SD	0		0.03 ± 0.07		0.03 ± 0.07		0.01 ± 0.03	
		Max	0		0.2		0.2		0.1	
		N	0		3		3		2	
	HA	Mean ± SD	0.02 ± 0.05		0.07 ± 0.18		0.04 ± 0.08		0.01 ± 0.03	
		Max	0.20		0.74		0.2		0.12	
		N	2	0.1868	5	0.3325	5	0.5228	1	0.6963

Values are presented as number (n), maximum value (max), or mean ± standard deviation (SD). The listed *P* values were calculated by using *t* tests. Abbreviations: CPB, cardiopulmonary bypass; HA, hemoadsorption; POD, postoperative day

## Discussion

Cardiac surgery is associated with an unpredictable activation of the immune system with an increase of pro-inflammatory cytokines as well as a decrease of anti-inflammatory cytokines, which is caused by blood contact with artificial surfaces and therefore is linked to adverse outcomes [17, 18]. In this (to our knowledge) first controlled study in patients undergoing on-pump cardiac surgery treated with the CytoSorb™ adsorber, no significant differences of pro-inflammatory cytokine levels were found. Even though a reduction of absolute levels within the first 24 hours after CPB is noticeable, no significant changes were observed. Given that we did not observe any adverse device-related side effects or differences in reduction of blood cells or albumin, our study shows that using the CytoSorb™ adsorber cartridge in a CPB circuit is technically feasible.

The indication for the CytoSorb™ adsorber is to reduce cytokine concentrations in various clinical situations with elevated cytokine levels and has been tested previously and demonstrated significant cytokine adsorption [19–21], an effect we could not reproduce in our patients. There may be numerous reasons for that outcome: First, we had a mean treatment time of 191 ± 56 minutes, which may be too short to allow a significant reduction of cytokine levels, although we did not find any correlation between cytokine peaks and treatment time. In all previously conducted studies [19–21] and case reports [12, 22, 23], the treatment time was at least 4 hours up to 4 days. In a CPB-porcine model with 5 hours of treatment time, also no effect in IL-6 or TNF-α has been found [24]. Secondly, we effectively observed a CPB-triggered immunoactivation and an increase in cytokines and inflammation markers after CPB and therefore after HA treatment. So it might be owing to a concentration-dependent adsorption of CytoSorb™ that we did not observe any HA of cytokines. Third, we may have expected a too optimistic effect and therefore we may have planned a too small sample size, a fact which is also shown by the observed strong inter-individual differences in the amounts of cytokine levels. But at the time of planning this pilot study, no clinically relevant data concerning our primary outcome were available. And, fourth, we included only the least sick cohort of patients undergoing cardiac surgery. Although we did not restrict the EuroSCORE levels of our included patients, our exclusion criteria resulted in a moderate preoperative risk for postoperative outcomes. In a recently published cohort analysis [25] representing more than 9,000 cardiac surgical patients operated at our center, we found similar demographics, so that the population we investigated represents in our opinion a cross-section of elective moderate-risk patients operated at our center.

We rarely observed the production of TNF-α in our patients and this goes hand in hand with the contentious role of TNF-α within CPB; although some studies have shown an increase, others have not [26]. Also, IL-1β has been found to be detectable in only a small proportion of patients with systemic inflammatory response syndrome and sepsis [27].

IL-10 is thought to downregulate cytokine production [27], and high concentrations have been observed in our patients. Our results follow a similar time course to the pro-inflammatory cytokines with an early peak and subsequently falling concentrations. Interestingly, we observed a slower decrease of postoperative IL-10 levels in the HA group and therefore a longer effect up to 48 hours postoperatively. Recently, higher IL-10 levels following cardiac surgery have been associated with a decreased risk of mortality [28]. Although we did not find a reduction in mortality, this arguable immuno-protective effect needs to be investigated further.

We also found significant time-dependent changes in *ex vivo* LPS-induced TNF-α release. Surprisingly, we had observed a lower stimulation rate in the HA group already before treatment. That could be based on an effect modification of comorbidities like diabetes mellitus (DM). There is good evidence that patients with DM are associated with an immunosuppressive condition and have poorer humoral response, including decreased cytokine production, and therefore have an increased susceptibility to infections [29–31]. Although there were no differences between both groups, the lowest levels of LPS-induced TNF-α release were found in HA-treated patients with DM. Additionally, it may be an effect of an associated, preoperative drug therapy such as metformin, aspirin, or statins, which we did not record. HMGB1 is associated with the inflammatory response after ischemia/reperfusion injury after cardiopulmonary bypass, and it has been shown that a reduction decreases the markers indicating cardiac damage. Also, elevated levels of HMGB1 have been correlated with the disease severity of heart failure [32–35]. However, we observed preoperative differences in HMGB1; therefore, it is not possible to interpret the results if there is a post-treatment difference. According to our LPS-induced TNF-α results, we cannot rule out that there is a hidden phenotypic difference between groups despite randomization that may be associated with higher levels of HMGB1 before CPB. But we think the effects of HMGB1 and HA on patients’ postoperative course should be investigated further.

Last, we observed neither any differences in our patients’ body composition nor any need of catecholamines or postoperative fluid balances. Therefore, we cannot conclude that there is less edema formation in the HA group. Unfortunately, we did not record systemic vascular resistance after CPB to assess that the HA group was more vasodilated.

A limitation of a study such as ours may be effect modifications owing to omitted or unobserved confounding risk indicators, although we included the most relevant risk indicators to rule out any systematic effect. However, we did not monitor the use of non-steroidal anti-inflammatory drugs preoperatively (e.g., aspirin), statins, or metformin, which may have anti-inflammatory effects [36, 37]. Another limitation of our study can be that our study design was not double-blinded, only blinded in patients. Although blinding in studies involving operative management and medical devices is rarely feasible, we do not think that would have affected the outcome. Additionally, owing to technical problems, we were not able to perform BIA in every patient, and we have to report that our BIA monitor is validated in healthy subjects up to only 66 years [16]. Last, a limitation of the study is that we did not observe the cytokine levels before and after the HA cartridge and therefore we cannot be sure what proportion of blood has been treated. We can only estimate that despite the small amount of 3 to 4 % total blood volume purified each minute, more than 99 % of the blood volume has passed through the HA device after our mean treatment time of 191 minutes.

## Conclusions

We claim that the use of the CytoSorb™ adsorber cartridge in a CPB circuit is technically feasible. We observed a longer-lasting anti-inflammatory effect of IL-10 in the HA group, which needs to be investigated further. We did not observe significantly relevant changes in the evolution of pro-inflammatory cytokines in patients treated with the CytoSorb™ adsorber device during CPB. However, we did not find any effects on our patients’ clinical outcomes, although future studies should endeavour to increase the sample size. By reason of several involved pathways in the complex pathogenesis of the inflammatory reaction to CPB, the inhibition of a single pathway may not achieve sufficient inhibition of the entire pro-inflammatory cascade to significantly improve clinical outcomes [38]. We found an inhomogeneous inflammatory response expressed by high inter-individual differences in cytokine levels between our patients. A greater homogeneity may be achieved by identifying those patients or procedures, which are triggering an exaggerated inflammatory response to CPB like patients with endocarditis, procedures on the aortic arch needing hypothermic cardiac arrest or transplant surgery. This hypothesis requires further investigations.

## Key messages

- Using the CytoSorb™ adsorber during cardiopulmonary bypass did not change patients’ perioperative course.
- Activation of cytokines due to cardiopulmonary bypass is inhomogeneous and shows high inter-individual differences between our patients. Patients with an exaggerated inflammatory response to cardiac surgery need to be identified.
- A longer-lasting anti-inflammatory effect of IL-10 was observed in patients who were treated with hemadsorption.
- The use and installation of the CytoSorb™ adsorber on CPB were technically feasible, and no adverse device-related side effects occurred.

## Abbreviations

ACT: Activated clotting time
aHTN: Arterial hypertension
AoCC: Aortic cross-clamp
AP: Angina pectoris
BIA: Bioelectrical impedance analysis
BIS: Bispectral index
BMI: Body mass index
CABG: Coronary artery bypass graft
CKD: Chronic kidney disease
COPD: Chronic obstructive pulmonary disease
CPB: Cardiopulmonary bypass
CPR: Cardiopulmonary resuscitation
CRP: C-reactive protein
ECMO: Extracorporeal membrane oxygenation
EuroSCORE: European system for cardiac operative risk evaluation
HA: Hemoadsorption
HMGB1: High-mobility group box 1
IDDM: Insulin-dependent diabetes mellitus
IL: Interleukin
LOS-ICU: Length of stay in the intensive care unit
LPS: Lipopolysaccharide
LVEF: Left ventricular ejection fraction
MCI: Myocardial infarction
NIDDM: Non-insulin-dependent diabetes mellitus
PAOD: Peripheral arterial obstructive disease
PCC: Prothrombin complex concentrate
PCT: Procalcitonin
POD: Postoperative day
TBW: Total body water
TNF-α: Tumor necrosis factor-alpha
